# Evaluating the impact of boiling and roasting on the properties of cashew nutshells (*Anacardium occidentale* L.) for biomass valorization

**DOI:** 10.1038/s41598-026-38464-0

**Published:** 2026-02-27

**Authors:** Tatiana Cruz, Juan Porras, Camilo Hernandez, Juan Pablo Casas-Rodriguez, Camilo Ayala-García, Oscar Alvarez, Alicia Porras, Alejandro Maranon

**Affiliations:** 1 Grupo de Diseño de Productos y Procesos (GDPP), Department of Chemical and Food Engineering, Universidad de los Andes, CR 1 18a 12, 111711 Bogotá, Colombia; 2 Sustainable Design in Mechanical Engineering Research Group (DSIM), Department of Mechanical Engineering, Escuela Colombiana de Ingeniería Julio Garavito, Autopista Norte AK 45 205 59, 111166 Bogotá, Colombia; 3 Structural Integrity Research Group, Department of Mechanical Engineering, Universidad de los Andes, CR 1 18a 12, 111711 Bogotá, Colombia; 4 Department of Design, Universidad de los Andes, CR 1 18a 12, 111711 Bogotá, Colombia

**Keywords:** Cashew nutshells, Roasting, Boiling, Lignocellulosic biomass, Physicochemical properties, Chemistry, Energy science and technology, Environmental sciences, Materials science

## Abstract

Cashew nutshells (CNS), a lignocellulosic byproduct from the industrial crop *Anacardium occidentale* L., are a renewable material that constitutes 75 wt.% of the nut yet remains underused due to limited understanding of its processing. Before shelling—and the generation of this biowaste—thermal pretreatments such as boiling and roasting are commonly applied, but their effects on CNS remain underexplored. This study assesses their impact on the physical, chemical, thermal, and spectral properties of CNS. Boiling increased cashew nutshell liquid (CNSL) retention, influencing physical properties such as color, moisture content, density, and porosity. Chemical analysis revealed a high extractive fraction (46 wt.%), along with high lignin content and moderate cellulose and hemicellulose levels. Thermal degradation profiles differed notably: boiled CNS showed peaks linked to CNSL decarboxylation, while roasted and defatted samples exhibited typical lignocellulosic behavior. Boiled CNS exhibited reduced crystallinity in X-ray diffraction analysis; however, after defatting, the crystallinity of both pretreated samples became comparable. These results demonstrate that industrial thermal pretreatments significantly affect CNS composition, particularly through changes in residual CNSL. The combined compositional and thermal characterization provides valuable insight to guide future applications of CNS in biomass valorization pathways, supporting its integration into circular bioeconomy strategies.

**Supplementary Information:**

The online version contains supplementary material available at 10.1038/s41598-026-38464-0.

## Introduction

Lignocellulosic biomass is generated from a wide range of agricultural and industrial processes, including the cultivation and processing of non-food crops and edible commodities. These residues—composed primarily of cellulose, hemicellulose, and lignin—are abundant, renewable, and offer significant potential as raw materials for the development of bio-based products^1^. In the context of circular bioeconomy strategies, lignocellulosic resources have gained attention as sustainable alternatives to fossil-based feedstocks, enabling the production of materials, fuels, and chemicals with reduced environmental impact^2^. Their valorization supports waste minimization, pollution reduction, and the transition to resource-efficient industrial systems.

A representative example of lignocellulosic biomass is cashew nutshells (CNS), a fibrous, non-edible byproduct generated during the processing of cashew nuts (*Anacardium occidentale L.*), an industrial crop cultivated widely in tropical regions such as Brazil, India, Vietnam, and West Africa^3^. The CNS, which encloses the edible kernel, accounts for up to 70% of the nut’s total weight and is typically discarded. In 2023, global cashew production exceeded 5 million tons, generating approximately 3.5 million tons of CNS^4^. Structurally, CNS is composed of lignocellulosic layers and naturally contains cashew nutshell liquid (CNSL), a phenolic-rich extract that is caustic and flammable^5^. The lack of industrial utilization of CNS, combined with its volume and potentially polluting nature, highlights the need to better understand its characteristics to enable sustainable valorization and reduce its environmental impact.

Cashew nut processing typically involves several stages. First, the entire fruit is harvested, and the nut is separated from the pseudo-fruit for sun-drying. Then, the whole nut undergoes thermal pretreatment—either boiling or roasting- which is essential for efficient kernel extraction due to the nutshell’s hardness and the presence of CNSL. After boiling or roasting, the nuts are shelled to extract the kernel, which is then dried, peeled, and graded for commercialization.

In this study, the term *pretreatment* refers specifically to the industrial thermal process applied prior to shelling to facilitate kernel extraction. The two most common thermal pretreatments are roasting and boiling^3^. Roasting involves immersing nuts in a high-temperature oil bath, where they fry in their own CNSL. This method makes the shell brittle, facilitating cracking while enabling CNSL recovery^6,7^. It is widely used in small-scale processing due to its low initial investment but generates toxic vapors, posing occupational hazards^8–10^. Boiling, in contrast, involves submerging nuts in boiling water or exposing them to pressurized steam to soften the shell. Although this method results in harder-to-open shells, it is preferred by large industries due to its lower emission of toxic vapors^11^. Nevertheless, this process generates wastewater containing CNSL, raising environmental concerns^12^. While previous studies have examined the effects of these methods on kernel quality and shelling efficiency^6,13,14^, limited information is available regarding their impact on CNS properties^15^.

Previous studies have characterized CNS in terms of ultimate and proximate composition, as well as thermal properties such as calorific value and thermogravimetric behavior. Additional research has investigated their physical and chemical characteristics^16–18^. While a study^15^ examined the effects of roasting and boiling on color and moisture content, comprehensive analyses connecting thermal pretreatments to a broader set of CNS properties remain limited, particularly those integrating physical, chemical, thermal, and spectral characterization in a comparative framework. Understanding these properties is critical for assessing the potential of CNS as a bioresource in material, energy, or chemical recovery applications. To address this gap, this study systematically analyzes CNS derived from boiling and roasting, aiming to clarify how pretreatment-induced compositional changes affect their physical, chemical, thermal, and spectral behavior. This work contributes to a broader understanding of CNS as a biomass resource and supports its integration in value-added applications and circular bioeconomy strategies.

## Materials and methods

### Materials

Cashew Nutshells (CNS), an agro-industrial by-product of cultivated *Anacardium occidentale* L., were sourced from small-scale industrial producers in the Vichada Department (Colombia). They were obtained directly from the processing facility immediately after industrial processing, without prolonged storage. The subsequent experimental phase was conducted under controlled laboratory conditions in Bogotá, Colombia.

The CNS corresponded to nutshells generated during the shelling of roasted and boiled cashew nuts. Shelling refers to the process of separating the cashew kernels from their hard outer shells, leaving the nutshells as the material for this study. Before shelling, all cashew nuts were thermally pretreated by roasting or boiling. These conditions represent common local and global industrial practices used prior to kernel extraction and were not modified for this study. CNS without thermal pretreatment are not generated within the standard industrial processing stream, as thermal pretreatment is required to enable kernel separation. The specific conditions applied by the producers for each method were as follows:

- Roasting process: Carried out in open air at atmospheric pressure for approximately 1.5 to 2.5 min (min) at 190–210 °C. In this process, the nuts were placed over sand to cool down and absorb residual CNSL; most of the CNSL remained on the sand or the roasting pan.
- Boiling process: Conducted by submerging the nuts in water at about 11 bar for 9–15 min at 185–192 °C. Similarly, in this process, the nuts were placed on a mesh to cool and dry, allowing the CNSL to drain freely.

**Fig. 1:**
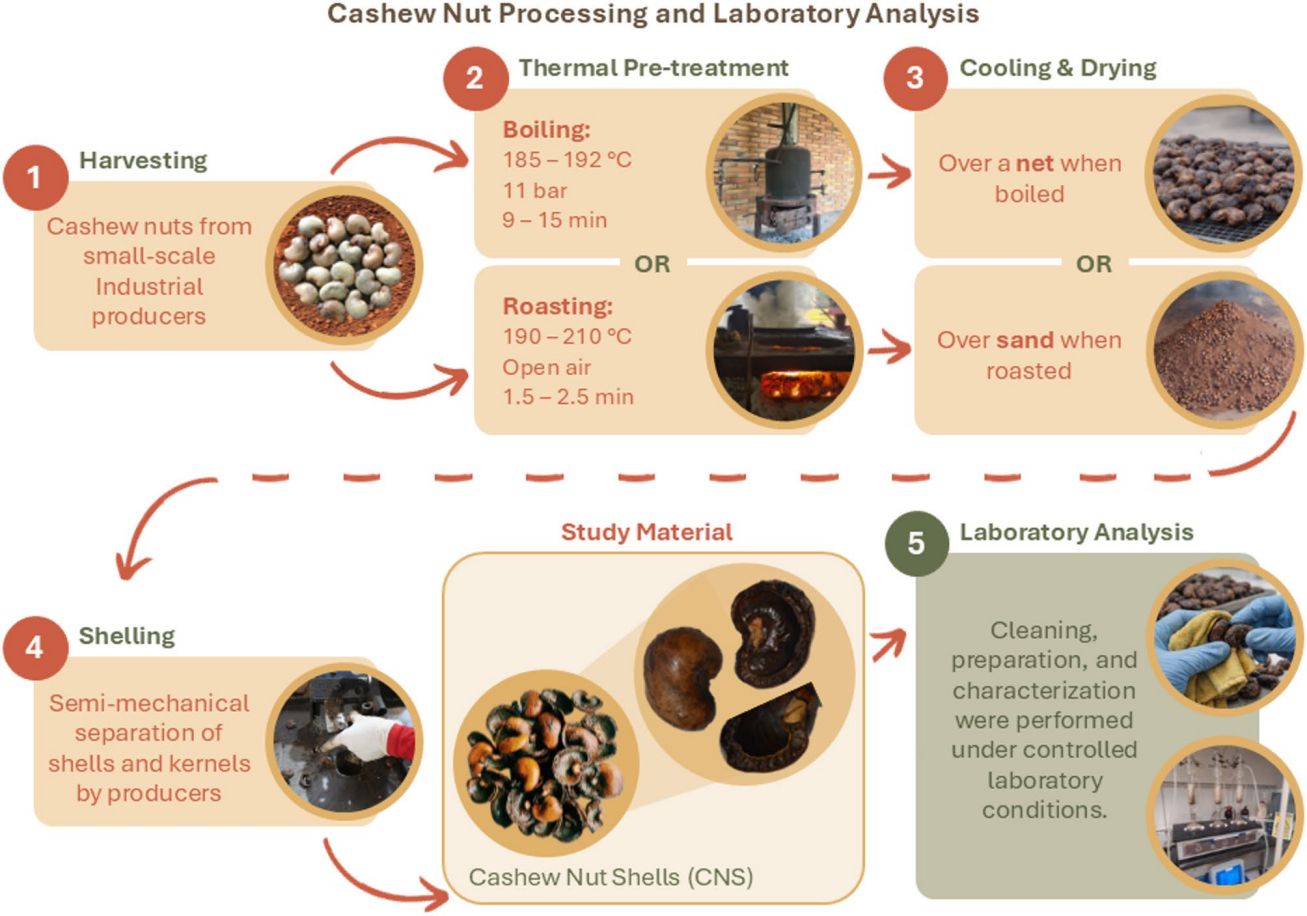
Overview of the workflow from industrial cashew nut processing to laboratory analysis of the resulting cashew nutshells.

Shelling was conducted using a mechanically assisted fixed-blade device operated by the producers. Once shelled and before testing, the CNS were cleaned with ethanol and cloth to remove dust and debris, although traces of CNSL still remained—a factor analyzed later in this work. Cashew nutshells obtained from roasting are referred to as CNS_R, while those from boiling are referred to as CNS_B. The overall processing workflow is summarized in Fig. 1.

To ensure random sampling for testing, multiple batches from each pretreatment group were thoroughly mixed within their respective pretreatment categories. Test samples from the roasted (CNS_R) and boiled (CNS_B) groups were then randomly selected using the quartering method^19^.

### Physical characterization

Physical characterization includes colorimetry, moisture content, density, and porosity assessment. Prior to analysis, cashew nutshells were selected to ensure comparable sizes, with widths ranging from 25 to 29 mm and lengths between 34 and 38 mm for all samples. These variables were selected to assess the effects of roasting and boiling pretreatments on the physical attributes of CNS. Color parameters provide insights into surface alterations associated with thermal degradation or chemical modifications; moisture content reflects water retention capacity; and density and porosity provide insights on the compactness and internal openness of the biomass resulting from heat exposure.

### Colorimetry

To determine the color attributes of the CNS surfaces, 30 samples from each batch (CNS_R and CNS_B) were analyzed following the method outlined by Madrid^20^ and ASTM D2244 standard. Color measurements were conducted using a Konica Minolta CR-20 color reader, calibrated with a white standard tile. The device was set to a 10° observation angle and illuminated by a D65 light source (corresponding to standard daylight, 6500 K). The CIELAB (L*, a*, b*) color space was used, where L* quantifies lightness (0 to 100 scale), a* represents red-green tones (positive for reddish, negative for greenish), and b* represents yellow-blue tones (positive for yellowish, negative for bluish)^21^.

For each sample, three random points on the surface were measured, and the average values of L*, a*, and b* were used. Additional parameters such as Chroma (C), which quantifies color intensity, and hue angle (H), which describes the perceived color type (e.g., reddish, greenish), were calculated. To compare CNS_R and CNS_B batches, differences in chroma (ΔC*), hue angle (ΔH*), and total color difference (ΔE*)—which represents the perceptible magnitude of color variation between samples—were also determined using standard CIELAB equations^21^ . Additionally, the variation in L*, a*, and b* was assessed to provide a more detailed comparison.

### Moisture content

The moisture content percentage of the CNS samples was determined following the ASAE S410.3 standard. Prior to moisture determination, CNS-R and CNS-B samples were conditioned for 24 h (h) at 20 ± 2 °C and 50 ± 5% relative humidity to ensure comparable initial moisture conditions.

For each pretreatment, three samples of approximately 200 g were oven-dried at 130 °C for 270 ± 5 min in a forced convection oven (Memmert UN450plus). Samples were considered dry when successive weight measurements taken at 30 min intervals showed no further mass change (constant mass condition). Moisture content was calculated using Eq. (1), where ${w}_{i}$ is the initial mass and ${w}_{f}$ is the mass after drying, measured with an accuracy of 0.1 g.

1 $$\% H = 1 - \frac{{w_{f} }}{{w_{i} }} \times 100$$

### Density and porosity

The true density (${\rho }_{t}$) of CNS_R and CNS_B was determined using the displacement method with ethanol at 20 ± 2 °C (ρ = 0.789 g cm^-3^), following the ASTM D792 standard, Method B. Prior to density measurements, CNS samples were conditioned for 24 h at 20 ± 2 °C and 50 ± 5% relative humidity. An analytical balance (VIBRA HT Series) with a precision of 0.1 mg, and an immersion set-up were used. Ten replicates were tested for each material.

On the other side, the bulk density (${\rho }_{b}$) of CNS_R and CNS_B was determined following the procedure presented by Kilanko^18^. A container with a known mass and volume was filled with randomly selected CNS samples and weighed. This measurement was repeated 10 times for each sample. The bulk density was then calculated using the following the equation:

2 $$\rho_{b} = \frac{mass of CNS in the container}{{Volume of the container}}$$

Finally, porosity (p) of the samples was calculated based on the known values of true ($\rho_{t}$) and bulk ($\rho_{b}$) densities, based on the following the equation proposed by Kilanko^18^:

3 $$p = \frac{{\rho_{t} - \rho_{b} }}{{\rho_{t} }} \times 100 \%$$

### Chemical composition

Chemical characterization was performed on three samples from each thermal pretreatment, following National Renewable Energy Laboratory (NREL) Biomass Analytical Procedures. Each of these samples was processed and analyzed independently for all chemical tests to ensure reproducibility (n = 3). The first step consisted of quantifying the extractive content of the CNS via Soxhlet extraction, following the procedure detailed in Sect. Extractive content. Prior to extraction, the CNS were dried and ground, then used for Soxhlet extraction. The biomass retained in the Soxhlet thimble was recovered and dried in an oven (Memmert UN450plus) at 105 °C. This defatted material was then used for the quantification of ash, lignin, and holocellulose content. Additionally, it served as the sample material for other characterizations, including spectral, thermal, and X-ray diffraction (XRD) analyses. The Soxhlet-defatted biomass is hereafter referred to as CNS_RD for roasted samples and CNS_BD for boiled samples.

### Extractive content

The quantification of extractives followed NREL/TP-510-42,619 standards. A Soxhlet apparatus was set up with 5.00 g (± 0.01 g) of dried and ground CNS (dry basis) and pre-weighed round flasks containing the selected solvent. The procedure consisted of two consecutive 24 h extraction phases: the first using distilled water as the solvent and the second using ethanol. After extraction, the round flasks containing water-soluble extractives were dried in the oven (Memmert UN450plus) at 105 °C for 12 h, while the round flasks containing ethanol-soluble extractives were dried in a rotary evaporator (YAMATO RE601) at 90 °C for 30 min. Once dried, the round flasks were weighed to calculate the extractive content of the samples.

### Ash content

The ash content in the CNS samples was determined in triplicate (n = 3) according to the ASTM E1755 standard. The crucibles were first cleaned and pre-weighed. Then, 1.00 g (+ 0.05 g, − 0.00 g) of each sample (CNS_RD and CNS_RB) were placed into separate crucibles. The crucibles were heated in a muffle furnace (Barnstead Thermolyne BenchTop) for 1 h at 300 °C followed by 4 h at 575 °C. After cooling to room temperature in a desiccator, the crucibles were weighed, and the percentage of ash content was calculated.

### Lignin content

Insoluble lignin was quantified following the NREL/TP-510–42,618 standard. For each run, 300 mg (-0 mg, + 10 mg) of the sample was weighed using a VIBRA HT Series balance and placed in a test tube. Then, a process of hydrolysis was carried out with 72% H2SO4 that involved stirring the test tube contents every 5 min and maintaining the tubes at a constant temperature of 30 °C. The contents of each test tube, along with 84 mL of Type I water, were transferred to several 250 mL Schott bottles, and autoclaved (TOMY SX-700) in a sterilization cycle for 1 h at 212 °C and at a pressure of 2 bars. After autoclaving, the samples were vacuum filtered using sintered glass crucibles (G3, 30 mL). The lignin retained the crucibles was then dried in an oven (Memmert UN450plus) at 105 °C for 12 h and weighed. To account for the structural ash in the biomass, the filtered lignin was subjected to the ashing procedure described in Sect. Ash content The measured ash content was then subtracted from the lignin weight to obtain the corrected lignin content.

### Holocellulose: hemicellulose and cellulose content

The quantification of holocellulose (cellulose and hemicellulose) followed the NREL/TP-510–42,618 standard. For this test, the hydrolysate obtained after lignin filtering was utilized. For each sample, 10 mL of hydrolysate was placed into Falcon tubes and neutralized using CaCO3, adjusting the pH to a range of 5 to 6. The neutralized Falcon tubes were centrifuged (Sorvall Legend XTR) at 4000 rpm to separate the calcium sulfate from the remaining liquid. The supernatant from each Falcon tube was then filtered through a 0.2 µm syringe filter and transferred to 1.5 mL vials.

High Performance Liquid Chromatography (HPLC) analysis was performed using the Bio LC Solutions 1260 Infinity III Bio-Inert LC system to determine the sugar contents. The analysis utilized a Bio-Rad Aminex HPX-87H column operated at 55 °C, with 0.005 M H_2_SO_4_ as the mobile phase at a flow rate of 0.6 mL/min. A refractive index detector (RID) was used, with an injection volume of 10 μL and a runtime of 20 min per sample. Glucose and cellobiose were quantified as cellulose markers, while xylose and arabinose served as hemicellulose indicators. Seven-point calibration curves were established for quantification, with concentrations ranging from 0.1 to 5 mg/mL. Galactose and mannose were not individually quantified due to insufficient chromatographic resolution from xylose under the selected HPLC conditions.

### Spectral analysis: Fourier transform infrared spectroscopy (FTIR)

FTIR analysis was performed to identify chemical functional groups and detect compositional changes qualitatively. The goal was to examine how roasting and boiling affect the presence or absence of characteristic bands associated with CNSL and lignocellulosic structures. A Thermo Fisher Scientific™ Nicolet™ iS50 FTIR Spectrophotometer® (Waltham, MA, USA) with Attenuated Total Reflectance (ATR) module was used. Each sample was analyzed over a wavelength range of 4000 and 400 cm^-1^.

Two specimens were evaluated for each surface of the boiled and roasted cashew nutshells, specifically: two from the internal (I) and two from the external (E) surface, designated as CNS_BI, CNS_BE, CNS_RI, and CNS_RE. Additionally, three measurements were performed on Soxhlet-defatted samples (CNS_BD and CNS_RD) for both boiling and roasting, and three measurements were conducted on CNSL. These latter three sample types served as references.

### Thermogravimetric analysis (TGA)

The objective of the TGA analysis was to identify the main mass loss regions of CNS samples and infer which components might be associated with each degradation event. Thermal behavior was evaluated for CNS_R, CNS_B, CNS_RD, CNS_BD and CNSL samples. TGA was performed on a TA Instruments SDT Q600 analyzer (New Castle, DE, USA) based on the ASTM E1131 standard. Samples were heated in a nitrogen atmosphere at a rate of 10 °C/min between 20–600 °C. This test established the decomposition temperature of CNS components. Each sample was analyzed twice under identical conditions to ensure result reliability.

### X-ray diffraction (XRD): crystallinity index

X-ray diffraction analysis was conducted to determine the crystallinity degree of CNS samples and to evaluate how this property is influenced by compositional differences among them, particularly after boiling or roasting. A Rigaku Ultima III x-ray diffractometer (Tokyo, Japan) was used at 40 kV and 40 mA with a monochromatic copper Cu Kα radiation source (λ = 0.1525 nm) in the step-scan mode, and a 2 $\theta$ angle ranging from 5 to 50° at rate of 1.5°/s. Two samples were analyzed per CNS type: CNS_R, CNS_B, CNS_RD, and CNS_BD.

The crystallinity index was calculated from each XRD profile based on the relative area of the crystallinity phase, following the procedure described in Choi and Chung^22^ and according to Eq. (4). This allowed a comparative assessment of crystallinity across the different CNS types.

4 $$\% C = \frac{crystalline phase area}{{total area XRD profile}} \times 100$$

### Statistical analysis

The influence of each pretreatment on moisture content, true density, bulk density, porosity, water- and ethanol-soluble extractives, lignocellulosic content, and crystallinity percentage was evaluated using a one-way analysis of variance (ANOVA) performed in Minitab 19 Statistical Software (Minitab Inc., State College, PA, USA). For each property, normality of the residuals and homoscedasticity were confirmed using the Shapiro–Wilk test and Levene’s test, respectively. Statistical significance was determined at a p-value threshold of less than 0.05. All results are reported as mean (standard deviation).

## Results and discussions

### Physical characterization

Results of physical characterization are presented below.

### Colorimetry

Colorimetry test was conducted to evaluate the impact of roasting and boiling on the appearance of CNS. The CIELAB parameters for each sample are shown in Table 1. According to the results, lightness (L*), redness (a*) and yellowness (b*) are greater in CNS_R than in CNS_B. CNS_R also shows higher color intensity (ΔC*) than CNS_B. The H* value indicates that both colors are in the same range, between reddish and yellowish. However, CNS_R are slightly yellower (ΔH*) than CNS_B. Finally, ΔE* is greater than 3, which means that the color difference between the two nutshells is perceivable by human eye as “very distinct”^21^.

**Table 1:** Colorimetry results.

Sample	L*	a*	b*	C*	H*	
CNS-R	38.05 (3.99)	10.60 (1.80)	13.53 (3.21)	17.19	51.93°	-
CNS-B	32.31 (3.80)	5.95 (1.79)	6.50 (3.06)	8.81	47.54°	-

	ΔL*	Δa*	Δb*	ΔC*	ΔH*	ΔE*
${\Delta }_{R-B}$	5.74	4.65	7.03	8.38	4.58°	10.20

Standard Deviation is reported between the parentheses.

The roasting process results in a light brown color of CNS, while boiling process generates a darker CNS. One possible explanation considered was partial thermal degradation of the lignocellulosic material, as reported in other natural fibers exposed to prolonged heating^23^. However, the darker appearance of CNS_B is more likely explained by the presence of residual CNSL on the surface.

### Moisture content

Experimental results (Table 2) revealed that CNS_B exhibited a higher MC than CNS_R, with a statistically significant difference (F = 7.67, p = 0.033). This difference may be attributed to boiling, which involves water exposure and increases the inherent MC of the processed CNS. Additionally, the residual CNSL on the surface of CNS_B may act as a partial barrier to moisture transfer, potentially contributing to the observed differences in moisture retention within the lignocellulosic biomass. Previous studies on ground CNS samples reported MC values ranging from 5.36^24^ to 9.83%^25^. The values obtained in this work fall within this range and are consistent with those reported in Table 2 for other nutshells.

**Table 2:** Physical properties of lignocellulosic byproducts.

Lignocellulosic Byproducts	Moisture content [%]	Bulk density [g cm^-3^]	Reference
CNS-R	6.21 (0.71)	0.23 (0.01)	This work
CNS-B	8.28 (1.31)	0.36 (0.01)	This work
Almonds shells	6.90	0.37	^27,28^
Walnuts shells	6.70	0.65	^27,28^
Pecans shells	10.4	0.64	^27^
Hazelnuts shells	9.56	0.55	^29,30^
Macadamias shells	9.20	0.68	^31^
Rice husk	8.59	0.10	^32^
Cocoa pod	10.96	0.71*	^33^
Coconut shells	10.33	0.69**	^34^

*Grounded biomass < 1 mm; **Grounded biomass < 12.5 mm; Standard Deviation is reported between the parentheses.

### Density and porosity

The true densities of two batches of CNS—CNS_R and CNS_B—were measured, indicating that CNS_B had a slightly higher density. The values obtained were 0.64 g cm^-3^ (0.02) for CNS_R and 0.69 g cm^-3^ (0.04) for CNS_B, with standard deviations in parentheses. Statistical analysis confirmed a significant difference between the samples (F = 8.79, p = 0.006).

Bulk density measurements confirmed that CNS_B also had a higher bulk density than CNS_R. Statistical analysis revealed a significant difference between the two samples (F = 3951.10, p = 0.000). These results, along with reference values from other lignocellulosic agro-industrial byproducts, are presented in Table 2. De Paula^26^ reported a bulk density of 0.254 g cm^-3^ for broken CNS, which closely aligns with the value obtained for CNS_R (0.234 g cm^-3^). Additionally, various studies have reported an average bulk density of 0.444 g cm^-3^ for ground CNS^24,25^; however, these values often lack information about the pretreatment used.

In summary, the observed density values reflect the impact of thermal pretreatments and are influenced by the presence of residual CNSL, retained within the alveolar structure and on the nutshell surface. Given that both batches had nuts of similar size, the increased weight and density of CNS_B can be attributed to the retention of CNSL during boiling, rather than to intrinsic differences in the original biomass.

Porosity, a parameter calculated from true and bulk densities, also showed statistically significant differences between the batches (F = 2650.84, p = 0.000). CNS_R exhibited higher porosity (63.35% (0.68%)) compared to CNS_B (48.20% (0.63%)). This reduction in porosity could be attributed to residual CNSL and moisture retention within the porous matrix of CNS_B. Additionally, boiling may induce some degree of structural compaction due to water absorption and softening of cell walls, a phenomenon less likely under dry-heat roasting conditions. Direct comparisons with other particulate lignocellulosic byproducts are challenging, as most reported bulk density values refer to ground biomass.

### Chemical composition

Table 3 presents the chemical composition of CNS for both CNS_R and CNS_B samples. The results indicated a similar content of water-soluble extractives between CNS_R and CNS_B. This suggests that pretreatment does not significantly (F = 4.21, p = 0.523) affect water-soluble extractives such as inorganic material, non-structural sugars, and nitrogenous materials. However, a statistically significant difference (F = 7.80, p = 0.049) exists in ethanol-soluble extractives between the two samples. These extractives primarily consist of lipids, resins, organic acids and phenolic compounds, mainly associated with CNSL components^35^. These findings are consistent with the physical characterization data and demonstrate that thermal pretreatments impact the ethanol-soluble fraction of CNS. This effect is likely associated with the final processing phase (“excess removal”), where sand use during roasting might facilitate the removal of CNSL components.

**Table 3:** Chemical composition of CNS.

Sample	H_2_O-soluble Extractives [%]	Ethanol-soluble Extractives [%]	Ash [%]	Lignin [%]	Hemicellulose [%]	Cellulose [%]
CNS-R	25.02 (1.86)	20.91 (1.05)	0.48 (0.03)	28.72 (1.15)	6.86 (2.12)	16.50 (0.60)
CNS-B	22.54 (0.97)	23.12 (0.88)	0.48 (0.06)	27.71 (0.88)	6.61 (0.63)	15.80 (0.74)

Standard Deviation is reported between the parentheses.

In particular, CNS exhibits large amounts of extractives, accounting for approximately half of its total composition (45.97%), primarily due to the presence of CNSL. Although physical characterization suggested a significant difference between the two samples, mostly because of residual CNSL, chemical characterization results revealed no statistically significant difference (F = 0.11, p = 0.756) in the total extractives content between CNS_R and CNS_B. This discrepancy could be explained by the similar residual CNSL content in internal layers between the two samples. Additionally, the ash content analysis revealed no statistically significant differences between the CNS_R and CNS_B samples (F = 0.00, p = 1.000). As shown in Table 4, ash content in nutshells is typically low.

**Table 4:** Mean chemical composition of CNS compared to other lignocellulosic byproducts.

Lignocellulosic Byproducts	Extractives [%]	Ash [%]	Lignin [%]	Hemicellulose [%]	Cellulose [%]	Reference
CNS	45.97 (0.92)	0.48 (0.05)	28.22 (1.07)	6.73 (1.41)	16.15 (0.71)	This work
CNSc	-	0.92 (0.05)	54.71 (1.07)	13.06 (1.41)	31.31 (0.71)	This work
Almonds shells	5.7	0.7	28.8	27.1*	23.7*	^45^
Walnuts shells	10.6	0.7	29.9	22*	21.7*	^45^
Pecans shells	12.6	2.3	29.4	18.7	32.3	^39,48^
Hazelnuts shells	18.8	0.5	32	23.1*	29.9*	^46,49,50^
Macadamias shells	6	0.2	40.9	16.6*	34.7*	^46,51^
Rice husk	14	20.1	16	20	30	^44^
Cocoa pod	23.7	10.8	34.0	12.0	30.4	^32^
Cocoa bean shell	14.4	9.1	22.7	14.7	42.2	^47^
Coconut shell	4.5	5.6	31.8	20.5	37.5	^44^

* Calculated from monosaccharide content; Standard Deviation is reported between the parentheses.

Lignocellulosic content showed no statistically significant differences between the CNS_R and CNS_B samples (F = 4.22, p = 0.109). The CNS values are notably lower than those of other nutshells (see Table 4). A more accurate comparison emerges when considering CNS cake (CNSc), the neat CNS lignocellulosic fraction after CNSL extraction, which revealed a predominance of lignin compared to other nutshells and lignocellulosic byproducts. This high lignin content affects the thermal and chemical behavior of the biomass, as it is associated with greater aromaticity and thermal stability^36,37^. These characteristics may favor certain valorization pathways—such as char production or the generation of phenolic-rich aromatics during pyrolysis—but may hinder performance in biochemical applications due to lignin’s resistance to microbial degradation^38^. Furthermore, lignin’s hydrophobic nature and limited compatibility with polymeric matrices pose challenges for its use in bio-based composites^39,40^. Understanding these trade-offs is key to selecting suitable pretreatment and conversion strategies within a biomass valorization framework.

CNS exhibited high lignin content, medium cellulose content, medium to low hemicellulose content, and very low ash content. These results are aligned with other references in literature, which report parameters within similar ranges: ash (0.70–2.75%)^41,42^, lignin (7.45–41.8%), hemicellulose (0.70% − 7.35%), and cellulose (11.50–64.57%^16,17,43^. However, the wide variability among literature values highlights the influence of methodological consistency in biomass chemical characterization.

In Table 4, CNS represents the average of all measured data, as previously mentioned there is no statistically significant difference among them. The CNSc values were calculated as percentages of the total biomass weight after the extraction of CNSL. The chemical composition values reported for the different lignocellulosic byproducts were obtained from the literature using standardized analytical methodologies; however, not all references employed identical procedures (e.g., gravimetric Van Soest^32,39,44^ or Klason-based methods^45–47^, or calculations derived from monosaccharide content), which should be considered when interpreting the comparative data.

### Spectral Analysis

**Fig. 2:**
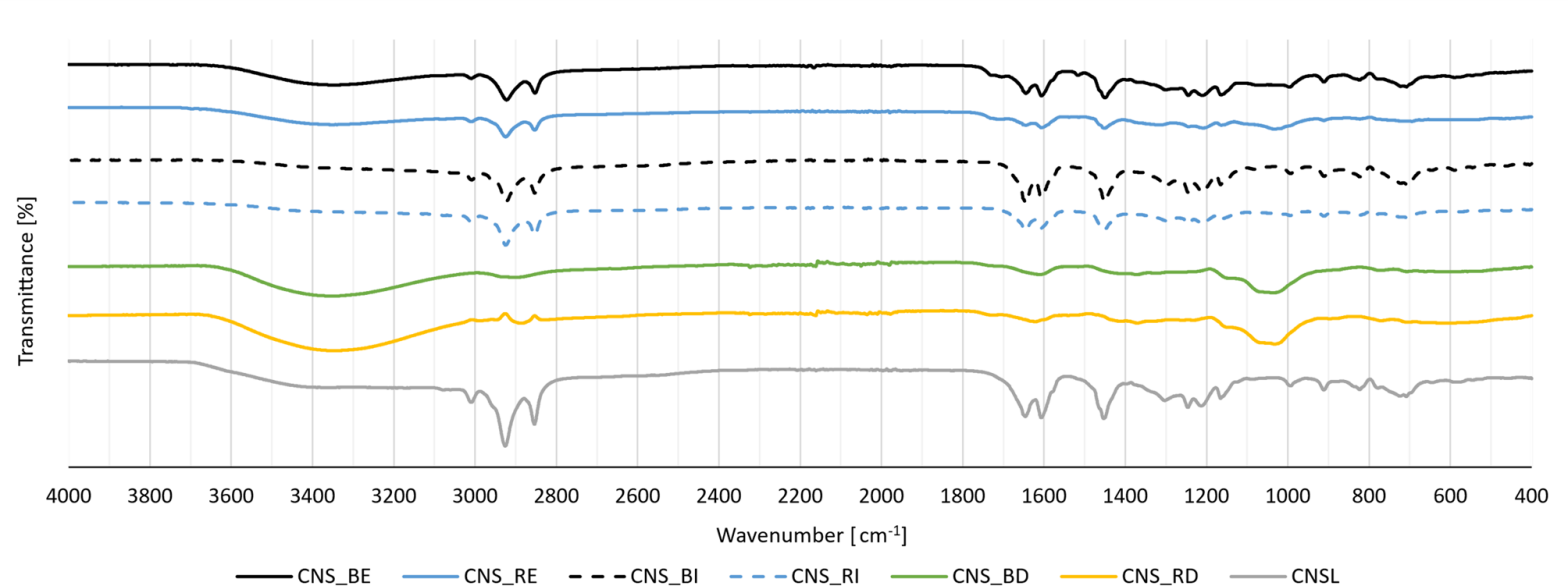
FTIR results for typical roasted and boiled cashew nutshells surfaces. Mixed scale.

FTIR analysis enabled the identification of functional groups present in the different CNS forms (see Fig. 2). The spectra of the external surfaces of both roasted and boiled cashew nutshells (CNS_RE and CNS_BE, respectively) revealed broad peaks in the range of 3700 to 3200 cm⁻^1^, attributed to O–H stretching vibrations associated with surface-bound water, phenolic or carboxyl groups from CNSL, and hydroxylic groups in lignin and polysaccharides. This band is less pronounced in the internal-surface spectra (CNS_BI and CNS_RI), which can be associated with the more compact and less porous microstructure of the inner shell (endocarp)^52^. Such a morphology may lead to lower surface area and reduced accessibility of hydrophilic sites, which may result in less intense O–H stretching bands, as reported for lignocellulosic biomaterials with reduced porosity^53^. In the defatted samples (CNS_BD and CNS_RD), the O–H band remains and is mainly associated with the exposed lignocellulosic fraction, which is more susceptible to moisture absorption.

Additionally, in the range of 2950 and 2850 cm^-1^, peaks corresponding to aliphatic C-H stretching vibrations were observed in CNS_RE and CNS_BE but disappeared in the defatted samples. The absence of these bands is attributed to the removal of CNSL components during the defatting process, as these bands are mainly associated with long aliphatic chains (–CH₂– and –CH₃) present in phenolic lipids such as cardanol, cardol, and anacardic acid. After CNSL extraction, the remaining lignocellulosic matrix lacks such long aliphatic chains, as these aliphatic C–H stretching vibrations are mainly associated with lipidic extractives rather than with structural lignocellulosic components^54^.

Peaks between 1750 and 1400 cm^-1^ are associated with double bonds, either C = C or C = O, and correspond to carboxylic acids and carbonyl groups found in CNSL components such as cardanol, cardol and anacardic acid^55^. These peaks were generally more intense in CNS_B than in CNS_R samples, possibly due to higher CNSL content or thermal decarboxylation of anacardic acid into cardanol or cardol induced by roasting.

These findings suggest that roasting and boiling markedly affect the functional groups on the CNS surface by reducing both the quantity and nature of residual CNSL. Internal surfaces of the nutshells, which were not exposed during thermal pretreatment, retained more CNSL and less moisture. In contrast, defatted samples showed the absence of characteristic peaks, confirming the effective removal of CNSL. These spectral changes are consistent with the chemical composition results, particularly the significant difference observed in ethanol-soluble extractives (CNSL-related compounds). The reduction of peaks attributed to C–H and C = O vibrations further supports the loss of initial CNSL components following pretreatment.

### Thermogravimetric analysis (TGA)

**Fig. 3:**
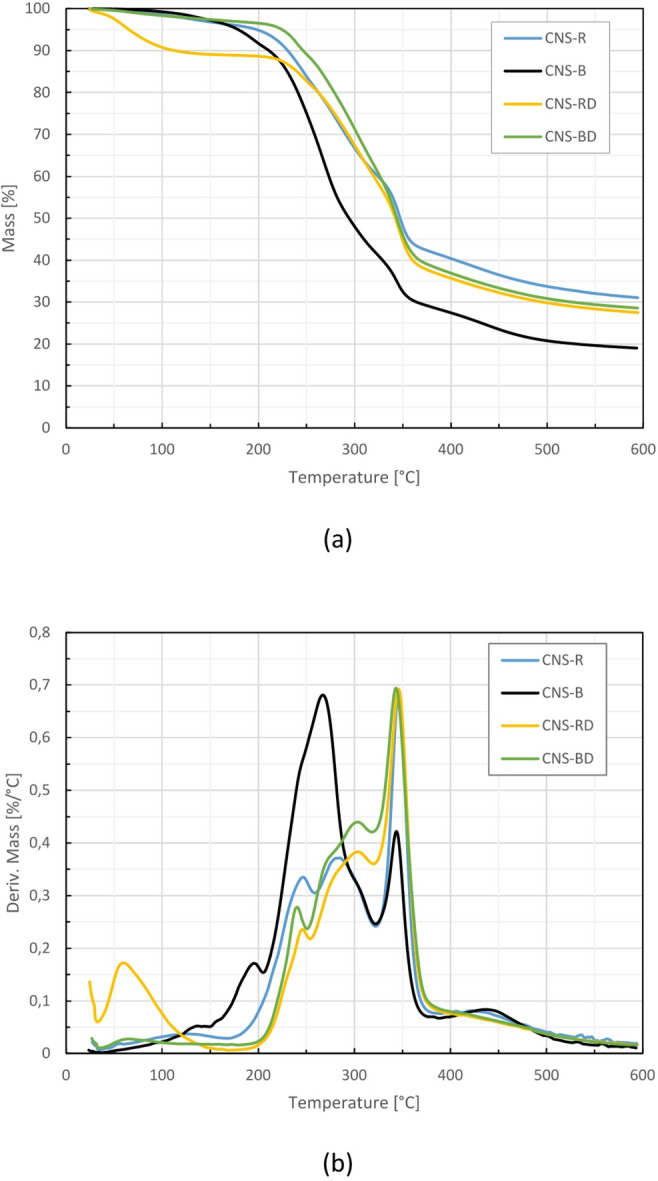
Thermal behavior of CNS: roasted, boiled, and after CNSL extraction. (**a**) TGA (**b**) DTGA.

The thermal stability of the CNS_R, CNS_B, CNS_RD, and CNS_BD samples was analyzed through TGA. The resulting weigh loss (TGA) and weight loss derivative (DTGA) curves are depicted in Fig. 3. According to the DTGA curve, the thermal degradation of the different CNS samples occurred in three distinct steps. The first step occurred from 20 to 220 °C. Below 100 °C, both CNS_RD and CNS_BD exhibited peaks associated with moisture loss. This may be because the defatted lignocellulosic fraction is more likely to absorb moisture.

In the case of CNS_B and CNS_R, observable peaks at 130 °C are attributed to both internal and external moisture loss^56^. This behavior occurred because traces of CNSL on the surface hindered the normal moisture evaporation at around 100 °C. Differences between moisture values obtained by oven-drying and TGA were observed and are explained by the longer exposure time in oven-drying, which promotes more complete evaporation. In contrast, TGA uses a continuous heating ramp, where water may not be fully desorbed before other thermal events begin.

To assess the influence of moisture on the comparison of thermal degradation behavior, the TG curves were additionally normalized by setting the residual mass at 110 °C to 100% (dry basis), assuming that the mass loss below this temperature is mainly associated with moisture and other low-boiling volatiles. The corresponding normalized TG and DTG curves are provided in Supplementary Information. After normalization, the TG/DTG curves of CNS-RD and CNS-BD show an almost complete overlap, reflecting comparable thermal degradation pathways consistent with their lignocellulosic composition. This normalization results in only minor changes in relative mass-loss values, while the main degradation steps and DTGA peak temperatures remain essentially unchanged.

Beyond the initial moisture-related events, a noticeable peak at around 180 °C was also observed for CNS_B, which has been linked to CNSL decarboxylation^57^. The higher amount of residual CNSL in this sample supports this interpretation and may also explain the nearly flat curves seen in the defatted samples between 150 and 200 °C.

The second step of degradation occurred between 220 and 350 °C (DTGA), with observable peaks for holocellulose and CNSL degradation^58^. In the case of CNS_R and the defatted CNS samples, DTGA displays peaks at approximately 245 °C, which are associated with hemicellulose degradation^59^. Around 300 °C, these samples presented a peak related to the initial stage of cellulose degradation^56^. However, for CNS_R, the peak is slightly shifted to the left, probably due to the degradation of residual CNSL at 280 °C^56,58^. Overall, CNS_R, CNS_RD and CNS_BD samples exhibit a typical lignocellulosic behavior^60^.

On the other hand, CNS_B presented a markedly different behavior from the other samples. The DTGA curve shows a broad peak at approximately 270 °C for this sample, representing the superposition of hemicellulose, initial cellulose and CNSL degradation^61^. This difference is attributed to the higher amount of residual CNSL in the CNS_B sample. This overlap highlights that thermogravimetric profiles alone may not provide a reliable estimation of lignocellulosic composition in CNS when significant amounts of residual CNSL are present, reinforcing the need for complementary chemical characterization. Finally, a second peak in cellulose degradation is observable at 345 °C for all samples^56,59^.

The last step of degradation reveals the remaining CNSL and lignin degradation occurring above 400 °C. Final CNSL degradation is particularly noticeable in the 430 °C peak for boiled and roasted samples^62^. In contrast, the lignin undergoes a wide-ranging degradation process that spans the entire profile, with its final degradation possibly extending up to 500 °C^59^.

Thus, the CNS samples exhibit a typical lignocellulosic degradation profile, characterized by distinct cellulose, hemicellulose, and lignin degradation peaks, which become more evident after the removal of CNSL. This profile is defined by a degradation temperature above 220 °C and a maximum degradation rate at 350 °C. The different pre-processing methods resulted in varying degradation behaviors in the CNS-R and CNS-B samples, attributed to differences in their CNSL content.

Conversely, the defatted samples exhibited similar performance regardless of the thermal pre-treatment, with CNS lignocellulosic fraction degradation temperature comparable to those of other natural fibers^63^. This suggests that defatted CNS could be incorporated in existing biomass valorization workflows, considering its thermal response and overall compositional profile. Moreover, the differences observed between roasted and boiled CNS highlight the relevance of the thermal pretreatment type when selecting biomass for specific valorization strategies, as residual CNSL may influence reactivity, processing behavior, or suitability for further transformation and utilization^57^.

### X-ray diffraction (XRD): crystallinity index

**Fig. 4:**
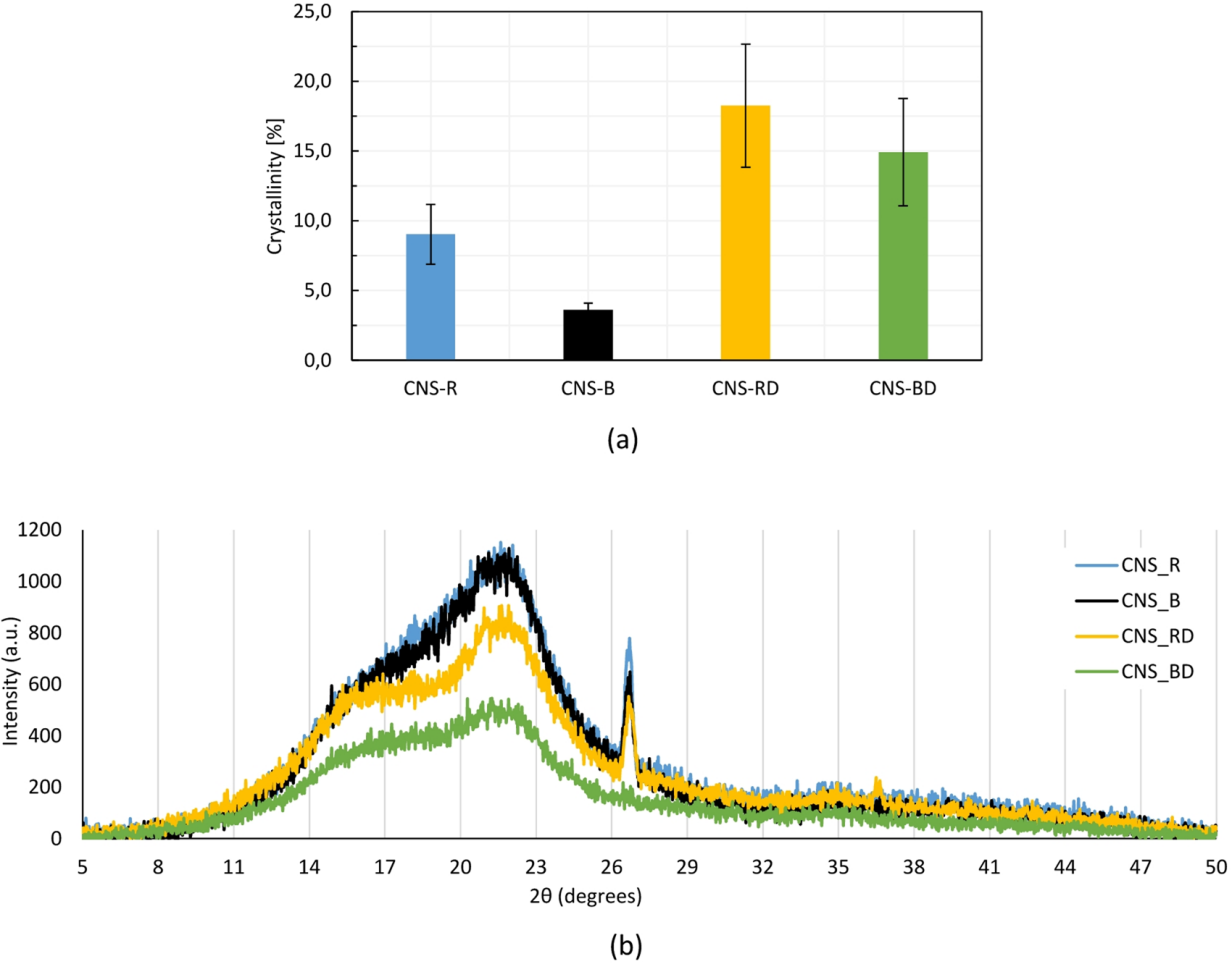
(**a**) Crystallinity index (%C), and (**b**) XRD spectra of roasted and boiled CNS, and their respective defatted samples.

Figure 4a. illustrates the crystallinity values of CNS samples pretreated by roasting and boiling, as well as their corresponding defatted samples. A statistically significant difference is observed between CNS_R and CNS_B (F = 18.29, p = 0.013), primarily due to variations in residual CNSL content. Conversely, CNS_RD and CNS_BD show no statistically significant difference in crystallinity (F = 0.96, p = 0.382), reflecting the removal of residual CNSL during defatting and yielding values representative of the true crystallinity of the lignocellulosic fraction.

A paired t-test was used to compare the crystallinity values of CNS_R and CNS_RD, as well as CNS_B and CNS_BD, because these comparisons involve dependent samples—the same material before and after the defatting process. Results revealed no significant difference in crystallinity between CNS_R and CNS_RD (T = 2.55, p = 0.125), likely due to the minimal residual CNSL in CNS_R, which had a negligible impact on crystallinity. However, a significant difference is observed between CNS_B and CNS_BD (T = 4.55, p = 0.045), consistent with the higher CNSL content in CNS_B^17,64^.

Figure 4b shows the XRD spectra of CNS samples. All samples exhibit a broad peak at 2θ ≈ 22°, typical of cellulose I, and a shoulder between 15 and 20°, commonly observed in lignocellulosic materials due to amorphous contributions from disordered regions of cellulose, lignin, and hemicellulose. Roasted and boiled samples display higher diffraction intensities, while defatted samples exhibit more defined peaks and higher relative crystallinity. These differences are attributed to the physical presence of CNSL within and on the surface of the CNS. CNSL, being an amorphous oily phase, elevates the background and can mask crystalline peaks, leading to an apparent reduction in crystallinity in samples with higher CNSL content. This interpretation is consistent with literature reporting that CNSL can be removed through purely physical extraction methods, such as solvent extraction or mechanical pressing, indicating a non-reactive interaction with the lignocellulosic fraction.

The crystallinity values, 18.24% (4.43%) for CNS_RD and 14.92% (3.84%) for CNS_BD, reflect the inherent crystallinity of the CNS lignocellulosic fraction. Notably, these values are up to 32% lower than those reported for other lignocellulosic byproducts, including almond nutshells (40%), walnut shells (49%), rice husks (48%), and corn cobs (32%)^65–67^. These findings align with the chemical characterization of CNS, where the relatively low crystallinity values reflect the influence of amorphous components like lignin and hemicellulose. This amorphous nature can enhance certain valorization pathways, including thermochemical processes (e.g., pyrolysis or hydrothermal liquefaction) where higher lignin content contributes to increased aromatic yields and char formation^68^. In contrast, in materials applications, amorphous components can influence interfacial bonding and overall performance when used in composites^69^. This combination of properties is relevant for biomass valorization, as it may enhance reactivity and conversion potential in thermochemical pathways or influence mechanical performance in material applications depending on the specific end use.

Considering these findings, the selection of CNS for specific valorization routes should account for factors such as processing conditions, residual CNSL content, and final application. Properties related to long-term stability, moisture interaction, or functional performance may become relevant depending on the context. Addressing these aspects in future application-driven studies would further support the targeted and responsible valorization of CNS-derived materials.

## Conclusions

This study demonstrated that boiling and roasting significantly influence the physical, chemical, thermal, and spectral properties of cashew nutshells (CNS). These effects are primarily driven by the variation in residual CNSL retention, which directly impacts parameters such as color, porosity, crystallinity, and thermal degradation behavior. Notably, while the integral shell properties vary depending on the pretreatment, the underlying lignocellulosic matrix—after defatting—exhibited a consistent composition. This indicates that the fundamental structural integrity of the biomass is preserved regardless of the industrial thermal processing applied.

In terms of valorization potential, the distinct characteristics of the pretreated samples offer versatile pathways. The boiled CNS, characterized by a high extractive fraction, shows suitability for thermochemical conversion processes such as pyrolysis or hydrothermal liquefaction for bio-oil recovery. Conversely, the thermal stability and moderate crystallinity of the defatted lignocellulosic fraction suggest it is an optimal candidate for use as a filler in composites or bio-based material formulations. Future research should focus on optimizing these specific integration pathways to fully support the inclusion of CNS in circular bioeconomy strategies.

## Supplementary Information

Below is the link to the electronic supplementary material.

Supplementary Material 1

## Data Availability

The data that support the findings of this study are available from the corresponding author upon reasonable request.
