# Supplementary material for: Evaluating the impact of boiling and roasting on the properties of cashew nutshells (Anacardium occidentale L.) for biomass valorization
Source: Sci Rep. 2026 Feb 27;16:11220. doi: 10.1038/s41598-026-38464-0 (PMC13046970; doi:10.1038/s41598-026-38464-0)
Supplement: Supplementary file 1 — Supplementary Material 1 [file 41598_2026_38464_MOESM1_ESM.pdf]

Supplementary Information

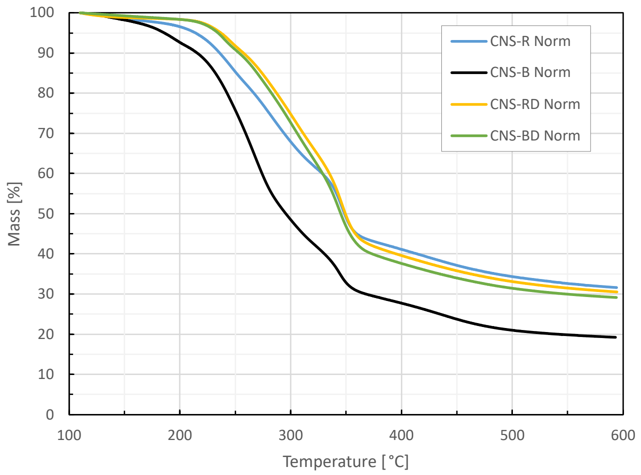

(a)

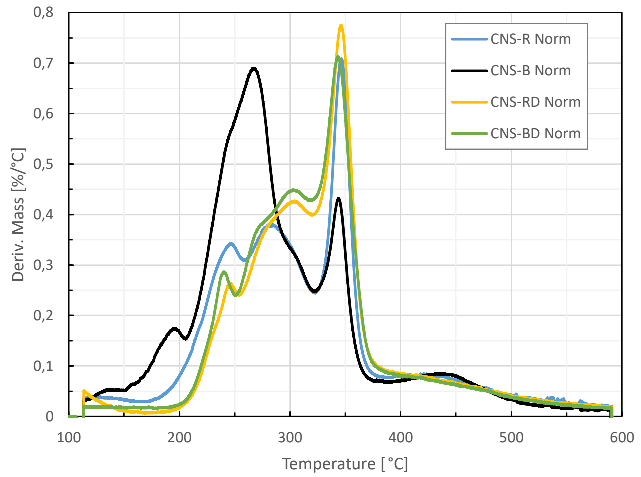

(b)

Figure S1. (a) Normalized thermogravimetric (TG) curves and (b) corresponding derivative thermogravimetric (DTG) curves of CNS-R, CNS-B, CNS-RD, and CNS-BD samples, normalized to a dry basis by setting the mass at 110 °C to 100%.

The TG curves were normalized by setting the residual mass at 110 °C to 100% (dry basis), assuming that the mass loss below this temperature is mainly associated with moisture and other low-boiling volatiles. DTG curves were recalculated from the normalized TG data using a centered finite-difference approach. Normalization results in only minor changes in relative mass-loss values, while the main degradation steps and DTG peak temperatures remain unchanged, confirming that the overall thermal degradation behavior is not significantly affected by residual moisture.
